# Preventative and therapeutic effects of a novel humanized anti-glycoprotein Ibα fragment of antigen-binding region in a murine model of thrombotic thrombocytopenic purpura

**DOI:** 10.1016/j.jtha.2025.02.009

**Published:** 2025-02-15

**Authors:** Liang Zheng, Reheman Adili, Zhijian Wu, Quan Zhang, Guangheng Zhu, Xi Lei, Zhenze Liu, Miguel A. D. Neves, Wenjing Ma, Sladjana Slavkovic, Xiaohong Ruby Xu, Heyu Ni, X. Long Zheng

**Affiliations:** 1 Department of Pathology and Laboratory Medicine, The University of Kansas Medical Center, Kansas City, Kansas, USA; 2 Institute of Reproductive and Developmental Sciences, The University of Kansas Medical Center, Kansas City, Kansas, USA; 3 Department of Laboratory Medicine, Keenan Research Centre for Biomedical Science, St. Michael’s Hospital, Unity Health Toronto, Toronto, Ontario, Canada; 4 Bloodworks Research Institute, Seattle, Washington, USA; 5 Department of Pulmonary and Critical Care Medicine, The Second Xiangya Hospital, Central South University, Changsha, China; 6 CCOA Therapeutics Inc, Toronto, Ontario, Canada; 7 Department of Laboratory Medicine and Pathobiology, University of Toronto, Toronto, Ontario, Canada; 8 Canadian Blood Services Centre for Innovation, Toronto, Ontario, Canada; 9 Department of Physiology, University of Toronto, Toronto, Ontario, Canada; 10 Department of Medicine, University of Toronto, Toronto, Ontario, Canada

**Keywords:** ADAMTS-13, glycoprotein Ib, monoclonal antibody, thrombotic thrombocytopenic purpura, von Willebrand factor

## Abstract

**Background:**

Thrombotic thrombocytopenic purpura (TTP) is a potentially fatal blood disorder resulting from severe deficiency of plasma ADAMTS-13 activity. Current treatment for immune-mediated TTP includes daily therapeutic plasma exchange plus caplacizumab and immunosuppressives. For hereditary TTP resulting from mutations of *ADAMTS-13*, plasma infusion or recombinant ADAMTS-13 is the treatment of choice. However, there are still unmet needs for an effective alternative therapy for TTP.

**Objectives:**

The present study aimed to evaluate the therapeutic efficacy of a novel humanized antibody fragment of antigen binding against platelet glycoprotein Ibα (CA1001) in a murine model of TTP.

**Methods:**

Platelet agglutination profiles, microfluidic shear-based assay, intravital microscopy thrombosis model, and lysine histone-induced murine “TTP-like” model were employed.

**Results:**

CA1001 exhibited potent inhibition of botrocetin-induced murine platelet agglutination in a dose- and time-dependent manner. CA1001 also significantly inhibited shear-dependent adhesion and aggregation of murine platelets to endothelial von Willebrand factor (VWF) released from calcium ionophore-activated cremaster venules in *Adamts-13* null mice and blocked the formation of platelet-VWF rich thrombosis. More importantly, CA1001 appeared to be efficacious in preventing and treating a histone-induced “TTP-like” syndrome in *Adamts-13* null mice, demonstrated by the alleviation of thrombocytopenia, prerenal injury, and formation of microvascular thrombosis in major organ tissues.

**Conclusion:**

CA1001 can effectively inhibit VWF-platelet interaction and thrombus formation under various (patho)physiological conditions. Thus, CA1001 may be a potential candidate for further development as a novel therapeutic for immune-mediated and hereditary TTP and perhaps for other inflammatory thrombotic disorders such as ischemic stroke.

## 1 | INTRODUCTION

Thrombotic thrombocytopenic purpura (TTP) is a potentially fatal hematological disorder resulting primarily from severe deficiency of plasma ADAMTS-13 activity [1]. The deficiency may be caused by mutations in *ADAMTS-13* [2] or the formation of autoantibodies against ADAMTS-13 protease [3]. Regardless of the cause of severe ADAMTS-13 deficiency, the end result is the formation of disseminated microvascular thrombosis, which leads to organ tissue damage [4]. ADAMTS-13 is the only plasma protease that could efficiently cleave newly released ultralarge von Willebrand factor (VWF) from activated or injured endothelium [5], although other proteases such as leukocyte protease, proteinase 3, cathepsin G may cleave VWF with relatively lower efficiency [6,7]. An inability to cleave VWF by ADAMTS-13 results in an accumulation of ultralarge VWF multimers on endothelial surfaces and/or at the sites of vascular injury, leading to excessive thrombus formation [8]. These large VWF/platelet-rich thrombi may be dislodged under flow and embolize the downstream small arterioles and capillaries, leading to the characteristic pathology of TTP.

Patients with TTP may present severe thrombocytopenia likely due to platelet consumption, microangiopathic hemolytic anemia, and signs and symptoms of organ ischemia and injury, including headache, confusion, stroke, chest pain, abdominal pain, and renal dysfunction [9]. The diagnosis of TTP relies on characteristic clinical signs and symptoms and laboratory tests, including complete blood count (CBC), peripheral blood smear, plasma or serum chemistry (eg, lactate dehydrogenase, creatinine, troponin I, haptoglobin, bilirubin, ADAMTS-13 activity and inhibitors, etc.), and radiographic imaging [10].

Based on the age of onset and plasma ADAMTS-13 activity and inhibitor test results, TTP can be classified into immune-mediated TTP (iTTP) if anti–ADAMTS-13 immunoglobulin (Ig) G or inhibitors can be detected and hereditary TTP if anti–ADAMTS-13 immunoglobulin G (IgG) or inhibitors are not detected on more than 1 occasion and confirmed by the findings of pathogenic mutations in *ADAMTS-13* [10].

Currently, all adult patients with a suspected TTP are treated emergently with daily therapeutic plasma exchange (TPE) plus caplacizumab and immunosuppressives (eg, corticosteroids and rituximab), known as triple therapy [11]. TPE may remove the IgG autoantibodies against ADAMTS-13 while replenishing the missing ADAMTS-13 protease from donor plasma [12]. Caplacizumab is a monoclonal nanobody that targets VWF at the A1 domain and inhibits VWF-platelet interaction, thus blocking thrombus formation despite ongoing severe ADAMTS-13 deficiency [13,14]. Immunosuppressives such as corticosteroids and rituximab block the formation of autoantibodies against ADAMTS-13 [15]. Such a triple therapy has significantly reduced the length of intensive care unit or hospital stay, decreased the number of TPE, and significantly reduced the mortality of acute iTTP [16–18].

However, there is still an unmet need for a potentially more efficacious and affordable alternative therapy for TTP. A strategy targeting the VWF-platelet interaction appears to have a direct and immediate effect on thrombus formation in TTP. For instance, caplacizumab binds the VWF A1 domain, which inhibits its binding to platelet glycoprotein Ib (GPIb)α [19]. However, this drug requires daily administration [14], and the cost of a course of therapy remains a concern [20]. Additionally, those with intracranial bleeding are contraindicated for caplacizumab [21]. Thus, targeting GPIbα directly may have some advantages because platelets have a much longer half-life than soluble VWF in circulation (3–4 days vs 8–12 hours) [22]. This may reduce the frequency of drug administration.

Here, we present the results of antithrombotic properties and therapeutic efficacy of a new humanized monoclonal antibody fragment of antigen binding region (Fab) against GPIbα (ie, CA1001) in a murine model of TTP. Our results demonstrate that CA1001 is a potent inhibitor of platelet-VWF interaction under various conditions. CA1001 appears to be efficacious in alleviating the disease severity in a histone-induced “TTP-like” syndrome in *Adamts-13* null (or *Adamts-13*^*−/−*^) mice.

## 2 | METHODS

### 2.1 | Animals

Mice (C57BL/6 and Balb/c) were used in the study according to the research protocols reviewed and approved by the Institutional Animal Care and Use Committees of the University of Kansas Medical Center, Kansas City, Kansas, USA, BloodWorks Research Institute, Seatle, Washington, USA, and the University of Toronto, Toronto, Ontario, Canada.

### 2.2 | Preparation of a novel humanized anti-GPIbα antibody

CA1001 was expressed in a single clone of stably transfected Chinese hamster ovary cells and purified by a series of purification processes using a good manufacturer practice (GMP) protocol. The purity of CA1001 reached 99.9%, assessed by sodium dodecyl sulfate–polyacrylamide gel electrophoresis and size exclusion high-performance liquid chromatography analysis.

### 2.3 | Western blotting

Following electrophoresis and transfer to a polyvinylidene difluoride membrane, a control IgG or purified CA1001 was detected with a horseradish peroxidase-conjugated goat anti-human κ chain antibody and a mouse anti-human κ chain antibody (GenScript), followed by chemiluminescent reagents (Thermo Fisher Scientific).

### 2.4 | Pharmacokinetic study

Mice (Balb/c) were intravenously (i.v.) administered with CA1001 at 0.5 mg/kg body weight. Blood samples were collected at various time points (up to 168 hours) following CA1001 administration. A volume of 10 μL blood from the saphenous vein was collected using a pipette and immediately transferred to 240 μL ethylenediaminetetraacetic acid solution for anticoagulation. The diluted plasma was obtained after centrifugation at 3000 rpm for 10 minutes. Plasma CA1001 levels were determined using an enzyme-linked immunosorbent assay. A mouse monoclonal anti-human κ chain unlabeled antibody (Southern Biotech) was used as the capturing antibody. Following blocking with 2% bovine serum albumin [23] at room temperature for 2 hours, diluted (1:4) plasma samples were added to the wells at 4 °C overnight. After washing, the bound CA1001 was detected by incubation with a horseradish peroxidase-conjugated goat polyclonal anti-human κ chain antibody (Southern Biotech) at room temperature for 1 hour. An o-phenylenediamine dihydrochloride peroxidase substrate (Thermo Fisher Scientific) was used for color development. The optical density was determined using a SpextroMax microtiter plate reader (Mol Devices). A purified CA1001 protein at various concentrations was used for calibration.

### 2.5 | Effects of CA1001 on botrocetin-induced platelet agglutination of murine platelets *in vivo*

Mouse whole blood was collected in sodium citrate from wild-type mice (Balb/c), and platelet-rich plasma (PRP) was isolated following centrifugation (500 × *g*). The PRP was incubated with various concentrations (0–20 μg/mL) of CA1001 for 30 minutes. Alternatively, CA1001 (1.0 mg/kg body weight) was injected into wild-type mice (Balb/c), and blood was collected at 0, 10, 30, 60, and 120 minutes following drug administration. The whole blood and PRP were prepared similarly to the one described above. The platelets in PRP were then activated with botrocetin (40 μg/mL, Sigma-Aldrich), and the rate of platelet agglutination or aggregation was recorded in a PAP-4 platelet aggregation profiler (Bio/Data Corporation) for 90 seconds [24]. The area under the curve was determined at the end of the experiments (seconds). Each experiment was performed at least 6 times.

### 2.6 | Effect of CA1001 on platelet adhesion and aggregation on the collagen surface under arterial flow

BioFlux microfluidic channels (Cell Microsystems) were coated with fibrillar collagen (Chrono-Log, 50 μg/mL) in 0.01 N hydrochloric acid (Sigma-Aldrich) for 2 hours. The surface was then blocked with 1% bovine serum albumin in phosphate-buffered saline. Whole blood was collected from adult *Adamts-13*^*−/−*^ mice (C57BL/6J) and anticoagulated with PPACK (D-phenylalanyl-L-prolyl-L-arginine chloromethyl ketone, Sigma-Aldrich). The platelets were stained with a fluorescein (FITC)-conjugated anti-mouse CD41 antibody (Thermo Fisher Scientific). The whole blood samples in the presence or absence of CA1001 (0.12 mg/mL) were then flowed over the collagen-coated surfaces under 15 dyne/cm^2^ for 3 minutes. Time-lapse digital images were captured every 3 seconds for a total of 3 minutes. The relative fluorescent intensity, indicative of platelet adhesion/aggregation, was determined offline using Montage software as previously described [25,26].

### 2.7 | Effect of CA1001 on calcium ionophore-induced microvascular thrombosis in mice

*Adamts-13*^−/−^ mice were injected with FITC-labeled donor murine platelets (1.25 × 10^9^ platelets/kg) of the same genotype as described in detail [27,28]. The mesenteric vessels were then exposed and treated with a drop of 10 μL calcium ionophore A23187 (Sigma-Aldrich) at 10 μmol/L to activate local endothelial cells to release ultra large VWF. The rate of platelet accumulation on the activated vascular surface was monitored under an intravital confocal fluorescent microscope. Time-lapse images were taken over a period of 20 minutes following the application of calcium ionophore with or without treatment of CA1001 (1.0 mg/kg body weight).

### 2.8 | Therapeutic efficacy of CA1001 in a murine model of TTP trigged by a lysine-rich histone

*Adamts-13*^−/−^ mice (C57BL/6J strain) at 4 to 5 months old were given a single i.v. injection of a lysine-rich histone (H5505) (Sigma-Aldrich) at 60 mg/kg body weight with or without i.v. administration of CA1001 (8 mg/kg body weight), followed by a subcutaneous (SQ) administration of CA1001 (4 mg/kg body weight) or vehicle control 4 hours after the H5505 challenge. Whole blood (70 μL) was collected via retro-orbital puncture prior to (−3 days) and 1 and 24 hours following the histone challenge for CBC analysis on a Hemavet950 (GMI). Residual blood was centrifuged at 3000 × *g* for 15 minutes, and plasma was collected for other biomarker analyses. Plasma levels of creatinine, blood urea nitrogen (BUN), and lactate dehydrogenase (LDH) were analyzed, as previously described [29].

### 2.9 | Histology and immunohistochemistry

Major organ tissues, including the brain, heart, lung, kidney, spleen, and liver, were obtained from mice after being sacrificed 24 hours after the H5505 challenge. The tissues were fixed with 4% paraformaldehyde in phosphate-buffered saline, paraffin-embedded, sectioned, and stained with hematoxylin and eosin. Additionally, immunohistochemistry was performed using a monoclonal anti-VWF antibody or anti-GPIIb/IIIa antibody as previously described [29,30]. Whole slides were scanned into digital images using the high-resolution scanner Zeiss Axioscan 7, and analyzed using ZEISS ZEN lite software. The number of thrombi in each slide section was counted manually.

### 2.10 | Statistical analysis

The Mann–Whitney U-test or one-way analysis of variance (ANOVA) was calculated with Prism 10 software (GraphPad). *P* values less than .05 and .01 were considered statistically significant and highly significant, respectively.

## 3 | RESULTS

### 3.1 | CA1001 inhibits botrocetin-induced platelet agglutination *in vitro*

**FIGURE 1:**
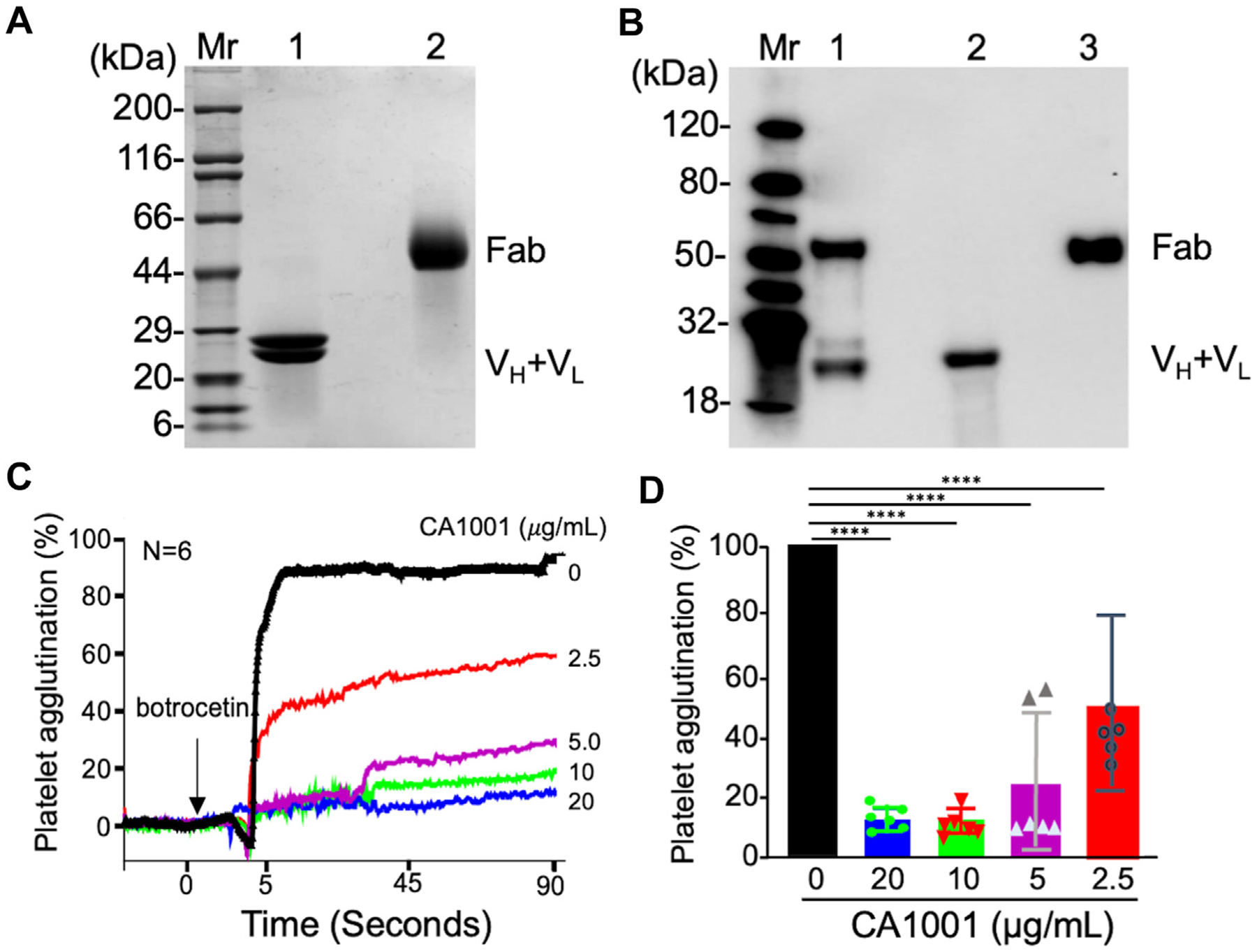
CA1001 inhibits botrocetin-induced agglutination of murine platelets *in vitro*. (A and B) Sodium dodecyl sulfate–polyacrylamide (SDS) gel electrophoresis stained with coomassie blue and probed with horseradish peroxidase (HRP)-conjugated goat anti-human immunoglobulin (Ig) G and a mouse anti-human IgG fragment of antigen binding region (Fab), respectively. Lanes 1 and 2 in panel A show the band(s) for purified CA1001 under denaturing/reducing and denaturing/nonreducing conditions, respectively. Lanes 1 in panel B is the control IgG under a denaturing/reducing condition, and lanes 2 and 3 are CA1001 under denaturing/reducing and denaturing/nonreducing conditions, respectively. (C) Representative tracing of agglutination of murine platelets in platelet-rich plasma that were stimulated with botrocetin (40 μg/mL) in the presence of an increasing concentration of CA1001 (0, 2.5, 5, 10, and 20 μg/mL) over 90 seconds. (D) The mean and standard deviation (SD) of the relative platelet agglutination or aggregation at the end of 90 seconds (*n* = 6). All results were normalized to the control of 100%. Kruskal–Willis analysis was performed to determine the statistical significance. of the various groups. ****P < .0001. V_H_ and V_L_ are the heavy and light chain of variable region, respectively.

To develop a novel therapy for thrombosis, we expressed and purified CA1001 using a GMP protocol. Following various optimization strategies, we purified recombinant CA1001 to homogeneity with >99.9% assessed by size exclusion high-performance liquid chromatography (HPLC). The purified CA1001 ran at ~25 kDa and ~50 kDa under denaturing and reducing conditions, respectively, and a denaturing but nonreducing condition on sodium dodecyl sulfate (SDS)–polyacrylamide gel electrophoresis stained with coomassie blue (Figure 1A). Western blotting with a mouse anti-human κ chain antibody confirmed the size of the purified CA1001 both under denaturing/reducing and denaturing/nonreducing conditions (Figure 1B). When purified CA1001 (0–20 μg/mL) was added to platelet rich plasma, the rate of botrocetin-induced platelet agglutination was significantly inhibited in a concentration-dependent manner (Figure 1C, D). These results demonstrate that CA1001 is a potent inhibitor of platelet-VWF interactions *in vitro*.

### 3.2 | CA1001 inhibits platelet-VWF interaction or thrombus formation *in vivo*

**FIGURE 2:**
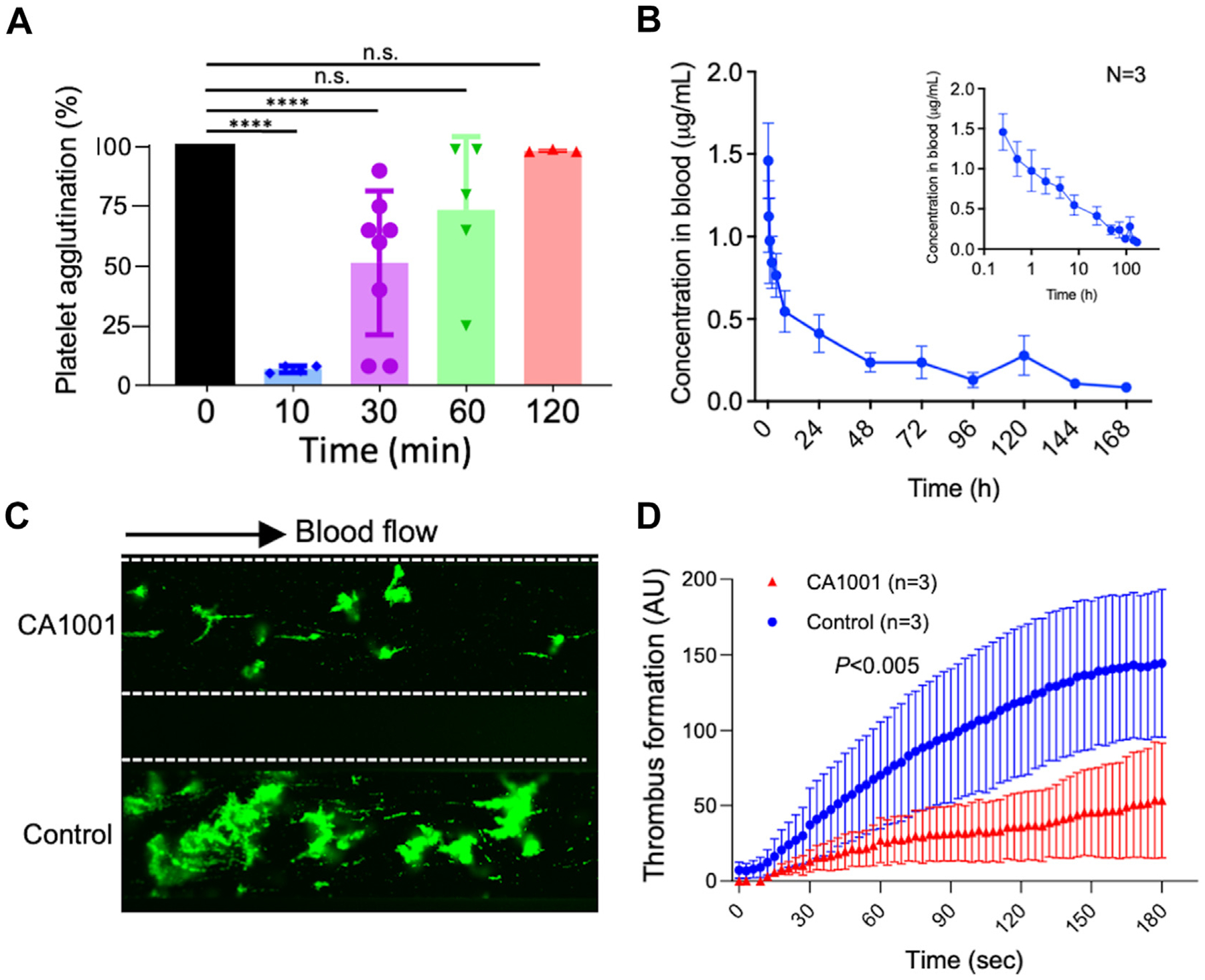
CA1001 reduces murine platelet agglutination and aggregation *ex vivo* or under flow conditions. (A) Botrocetin-induced agglutination of murine platelets in the platelet-rich plasma from mice at 10, 30, 60, and 120 minutes following an intravenous administration of CA1001 (1.0 mg/kg body weight). The data presented are individual values (dots), means (bars), and standard error of the means (SEMs) (horizontal lines). ****P < .0001; n.s., *P* > .05. (B) Half-life assessment for CA1001 in mice following an intravenous administration of CA1001 (0.5 mg/kg body weight). Plasma levels of CA1001 were determined by an enzyme-linked immunosorbent assay as described in the Methods. The inset shows the relationship between CA1001 levels over time in the log_10_-fold scale. The data shown are the mean and SEMs of 3 independent experiments (*n* = 3). (C and D). The final coverage and the rate of murine fluorescein-labeled platelet adhesion and aggregation over time, respectively, to a fibrillar collagen-coated surface under 15 dyne/cm^2^ following perfusion of the whole blood samples of *Adamts-13*^*−/−*^ mice with or without the addition of CA1001 (0.12 mg/mL) *ex vivo*. The data presented are the means and standard deviations (SDs) of 3 independent experiments (*n* = 3). *P* < .005 indicates that the difference between the CA1001- and vehicle-treated groups is statistically highly significant. AU, arbitrary units.

To assess the *in vivo* effect of CA1001 on platelet adhesion and aggregation, mice were injected i.v. with CA1001 (1.0 mg/kg body weight). Whole blood was collected from mice prior to and at 10, 30, 60, and 120 minutes following the CA1001 injection for a platelet aggregation assay. As shown, the percentage of botrocetin-induced platelet agglutination (the area under the curve) was significantly inhibited by CA1001 in a time-dependent manner (Figure 2A). The platelet reactivity appeared to be restored 2 hours following the i.v. administration of CA1001 at this dosage, consistent with a relatively short half-life (~4 hours) of CA1001 in mice following a single i.v. administration assessed by an enzyme-linked immunosorbent assay (Figure 2B).

To assess the efficacy of CA1001 on thrombus formation in mice with severe *Adamts-13* deficiency, we performed shear-dependent adhesion and aggregation of platelets from *Adamts-13*^*−/−*^ mouse whole blood on a collagen surface using a microfluidic assay. Whole blood samples were obtained from *Adamts-13*^*−/−*^ mice and anticoagulated with PPACK (100 μM). Following incubation without or with CA1001 (0.12 mg/mL), the blood samples flowed over a fibrillar collagen-coated surface under arterial shear (15 dyne/cm^2^). The final coverage (Figure 2C) and the rate of accumulation (Figure 2D) of FITC-labeled platelets onto the collagen-coated surface in CA1001-treated mice were dramatically reduced compared with those in the vehicle controls (*P* < .0001). These results demonstrate that CA1001 is a potent inhibitor of platelet-VWF interaction *in vivo*.

### 3.3 | CA1001 effectively inhibits calcium ionophore-induced microvascular thrombosis in mice

**FIGURE 3:**
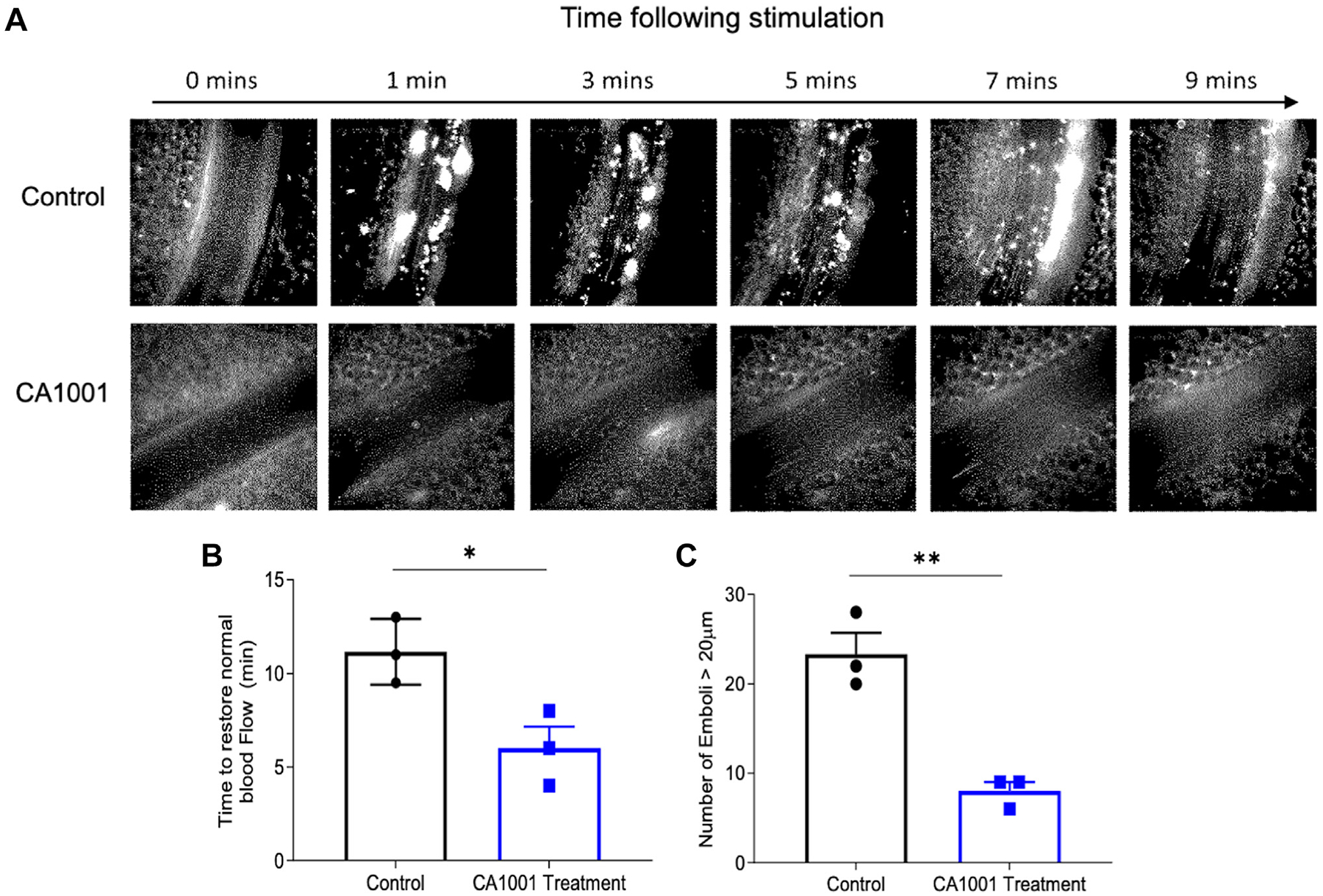
CA1001 effectively inhibits microvascular thrombosis in cremaster venules in *Adamts-13*^*−/−*^ mice. (A) Representative images showing the time-dependent accumulation of fluorescein-labeled platelets onto calcium ionophore-stimulated cremaster venules of *Adamts-13*^*−/−*^ mice treated without or with an intravenous administration of CA1001 (1.0 mg/kg body weight). (B) The time to restore normal blood flow and the (C) number of large thrombi (>20 μm) in the cremaster venules of *Adamts-13*^*−/−*^ mice treated without or with intravenous administration of CA1001 (1.0 mg/kg body weight). The data are shown as individual values, means (bars), and standard deviations (SDs) (horizontal lines). *P < .05; **P < .01.

Calcium ionophore activates endothelial cells and platelets, resulting in the release of endothelial VWF and inducing platelet adhesion, aggregation, and thrombus formation [31,32]. Using intravital microscopic imaging analysis, we found that pretreatment of CA1001 (1.0 mg/kg body weight) in C57BL/6 mice significantly reduced the accumulation of FITC-labeled platelets on the activated endothelial wall of mesenteric venules (Figure 3A and Supplementary Video S1). The time to restore normal blood flow following thrombus formation (Figure 3B) and the number of large thrombi (>20 μm; Figure 3C) was also significantly reduced with the administration of CA1001 compared with the vehicle-treated control. These results indicate that i.v. administration of CA1001 is efficacious in reducing acute thrombus formation resulting from vascular injury.

### 3.4 | CA1001 is efficacious in alleviating histone-induced TTP in a murine model

**FIGURE 4:**
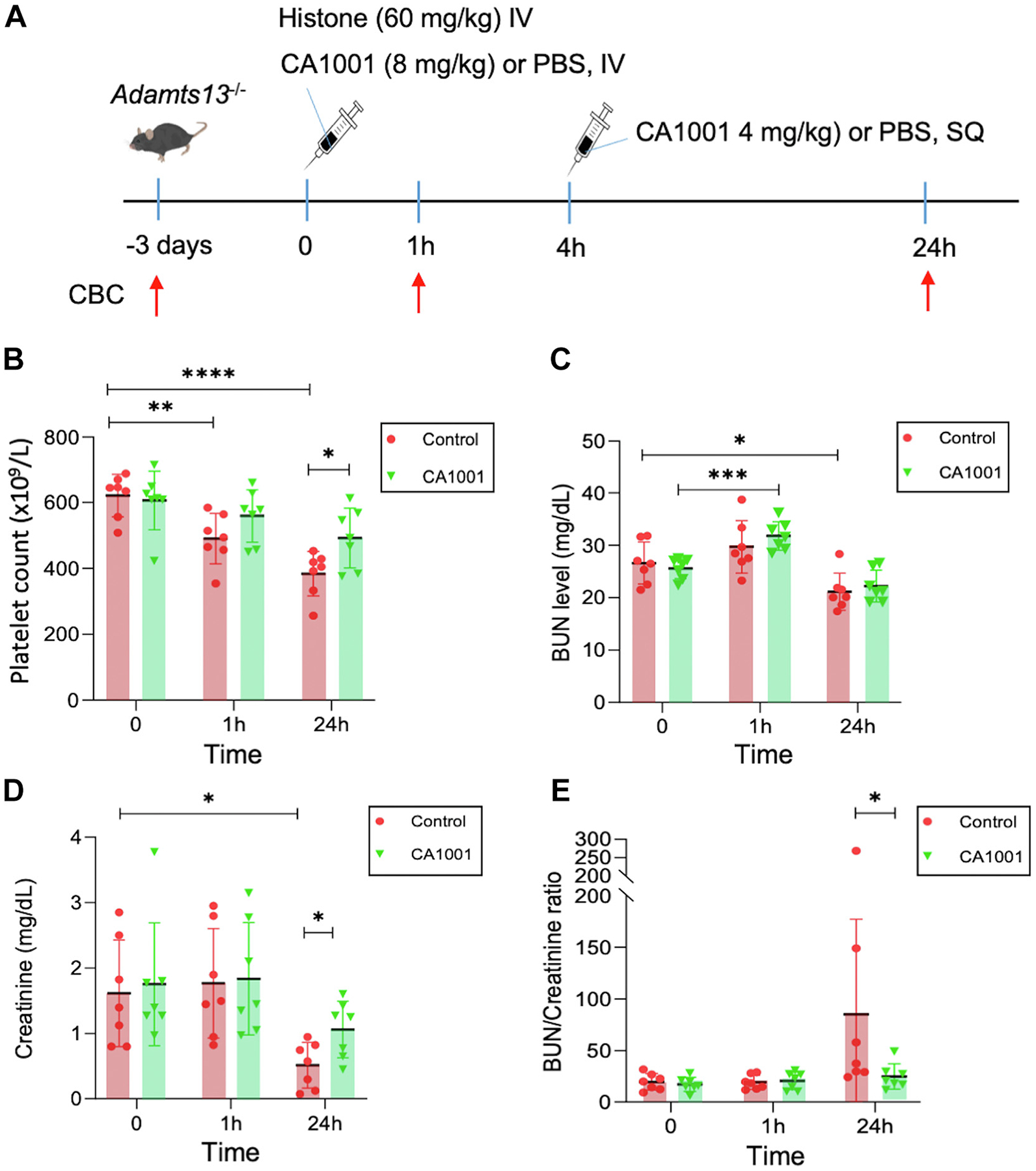
CA1001 alleviates histone-induced thrombocytopenia and prerenal injury in *Adamts-13*^*−/−*^ mice triggered by lysine-rich histone (H5505). (A) A schematic protocol for triggering the “TTP-like” syndrome in *Adamts-13*^*−/−*^ mice using H5505 with or without the administration of CA1001. (B) Platelet counts at 0, 1, and 24 hours in *Adamts-13*^*−/−*^ mice after being challenged with H5505 (60 mg/kg body weight) without or with the administration of CA1001 (8 mg/kg body weight, intravenously [IV] initially, followed by 4 mg/kg body weight, subcutaneously [SQ]). (C, D, and E) Plasma levels of BUN, creatinine, and blood urea nitrogen (BUN)/creatinine ratio at 0, 1, and 24 hours in *Adamts-13*^*−/−*^ mice after being challenged with H5505 without or with the administration of CA1001 as scheduled in panel A. Statistical analysis was determined using ANOVA with Prism 10 software. *P < .05; **P < .01; ***P < .005; and ****P < .0001 comparing histone challenge and CA1001 treatment. CBC, complete blood count; PBS, phosphate-buffered saline.

*Adamts-13*^*−/−*^ mice (C57BL/6J) do not develop spontaneous thrombocytopenia unless challenged with a bacterial Shiga toxin [33] or a large dose of recombinant VWF [34]. H5505 was shown to stimulate the rapid release of VWF from endothelial cells [35], which has been used to trigger an acute episode of TTP in an *Adamts-13*^*−/−*^ zebrafish model [30]. Here, *Adamts-13*^−/−^ mice were challenged with H5505 (60 mg/kg body weight) and were treated i.v. with CA1001 (8.0 mg/kg body weight) or a vehicle, followed by a s.c. administration of CA1001 (4.0 mg/kg body weight) or vehicle control 4 hours after the histone challenge, as illustrated (Figure 4A). CBC was performed 3 days prior to the histone challenge and 1 and 24 hours following the histone challenge with or without CA1001 treatment. As shown, the platelet counts in the vehicle-treated group dropped progressively, while the platelet counts in the CA1001-treated mice remained stable for 24 hours after the histone challenge (Figure 4B). The average platelet count in the CA1001-treated group was statistically significantly higher than that in the vehicle-treated controls (*P* < .05). Interestingly, there was a temporary increase in BUN in both the control and CA1001-treated mice 1 hour after the histone challenge, which returned to normal within 24 hours (Figure 4C). Plasma creatinine levels showed no significant change within an hour but were paradoxically reduced in 24 hours from the baseline after the histone challenge in both control and CA1001-treated mice. The reduction of plasma creatinine levels appeared to be greater in the control mice than in the CA1001-treated mice (*P* < .05; Figure 4D). However, the BUN/creatinine ratio, a better biomarker for assessing renal blood flow and perfusion conditions [36], was significantly increased in the vehicle-treated group but not in the CA1001-treated group (Figure 4E). These results indicate that CA1001 treatment may prevent and treat severe thrombocytopenia and ameliorate prerenal injury following the histone challenge in *Adamts-13*^*−/−*^ mice.

**FIGURE 5:**
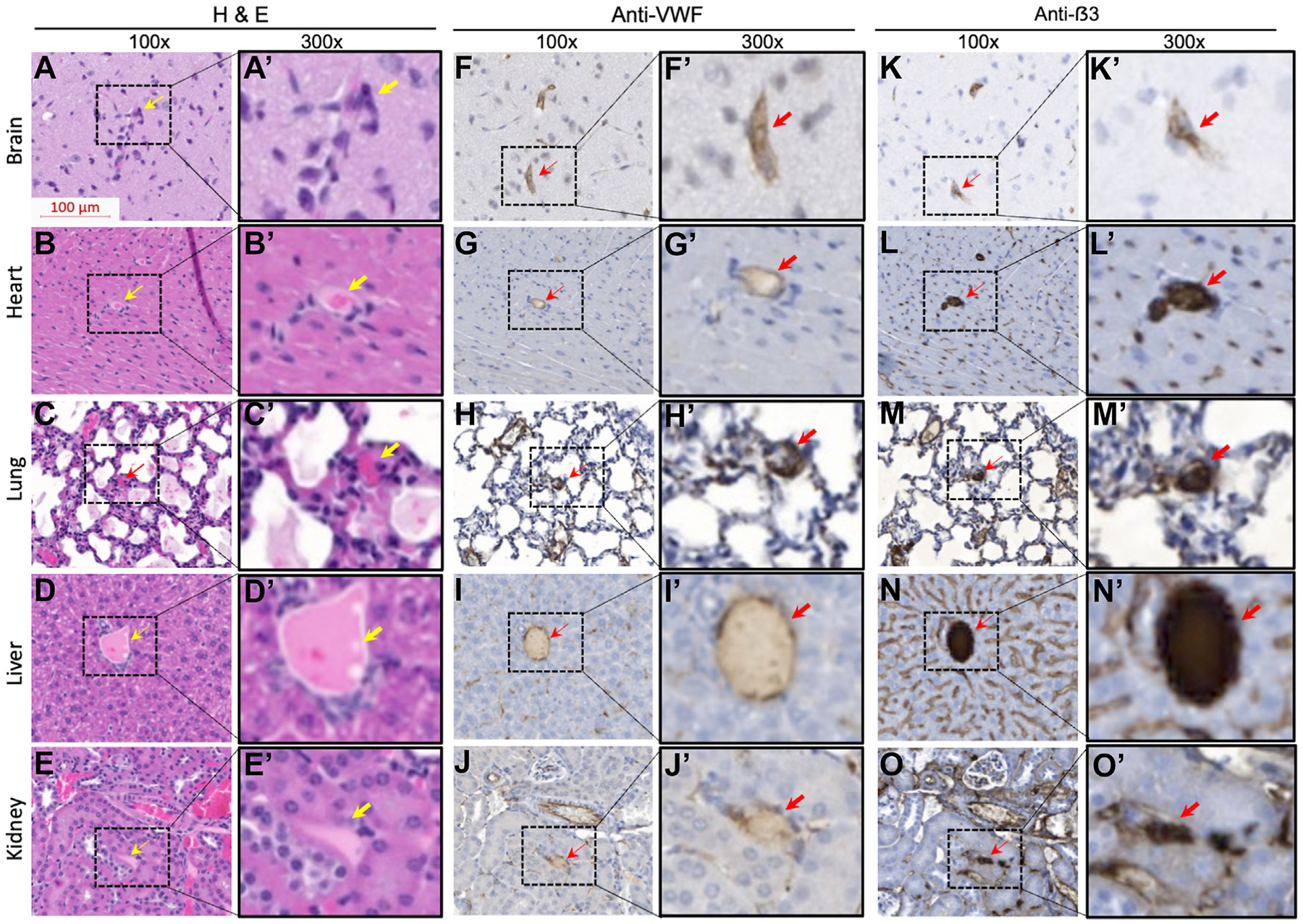
Histologic and immunohistochemical analysis of rare microvascular thrombosis in murine organ tissues. Representative images of tissue morphology of the brain, heart, lung, liver, and kidney from *Adamts-13*^*−/−*^ mice euthanized at 24 hours after a lysine-histone challenge without treatment of CA1001 stained with (A–E, 100× and A’–E’, 300×) hematoxylin and eosin (H&E), (F–J, 100×, F’–J’, 300×) anti-von Willebrand factor (Anti-VWF) immunoglobulin (Ig) G, and (K–O, 100×, K’–O’, 300×) anti-glycoprotein β3 (Anti-β3) immunoglobulin G. The arrows indicate the presence of an occlusive microvascular thrombus.

Histology and immunohistochemistry were performed in major organ tissues, including the brain, heart, lung, liver, and kidney. As shown, rare (0–2 per 10× lower field) occlusive microvascular thrombi were revealed by hematoxylin and eosin (Figure 5A–E, A’–E’) and immunohistochemical staining with antibodies against VWF (Figure 5F–J, F’–J’) or platelet GPβ3 (Figure 5K–O, K’–O’) in all tissues examined from the vehicle-treated mice 24 hours after the lysine-histone challenge, consistent with the diagnosis of mild disease of TTP. The prevalence of microvascular thrombi was significantly reduced in the liver and kidney. There was no significant difference in rare occlusive thrombi in the brain, heart, and lung in the *Adamts-13*^*−/−*^ mice following the histone challenge either being treated with vehicle or CA1001. These results indicate that lysine-histone may trigger a transient and mild “TTP-like” syndrome with evidence of rare VWF and platelet-rich microvascular thrombi. An i.v. infusion of CA1001, followed by a subcutaneous administration of CA1001, appears to reduce the formation of microvascular thrombosis in the liver and kidneys, where the endothelial VWF release may be most predominant following the i.v. histone challenge.

## 4 | DISCUSSION

The present study demonstrates the antithrombotic effect of a new humanized Fab fragment against platelet GPIbα (ie, CA1001) *in vitro* and *in vivo*. CA1001 may efficaciously ameliorate the disease severity (eg, thrombocytopenia and renal injury) in a murine model of “TTP-like” syndrome.

TTP primarily results from acquired autoantibodies that inhibit ADAMTS-13 activity and/or accelerate the clearance of ADAMTS-13 protease [37–39]. Rarely, TTP can also be caused by mutations in *ADAMTS-13* that result in secretion defects or impaired function of ADAMTS-13 [2]. The deficiency of plasma ADAMTS-13 activity leads to an accumulation of newly released ultralarge VWF that spontaneously attracts and agglutinates circulating platelets, forming occlusive thrombi in the microvasculature [40,41]. Current therapy for iTTP with TPE aims to remove autoantibodies against ADAMTS-13 and their immune complexes from circulation while replenishing the missing ADAMTS-13 enzyme from donor plasma. Adjunctive immune therapy with corticosteroids and rituximab (anti-CD20) reduces anti–ADAMTS-13 autoantibody production, which takes weeks to months to restore plasma ADAMTS-13 activity and see the therapeutic effect (eg, the recovery of platelet count, reduction of serum lactate dehydrogenase level, recovery of plasma ADAMTS-13 activity, etc.) [15]. A novel targeted therapy, such as the use of caplacizumab, an anti-VWF nanobody [13,14], aptamer, an anti-VWF A1 RNA molecule [42–44], or anfibatide, a natural antagonist of platelet GPIb isolated from snake venom [24,26], may stop thrombus formation despite ongoing severe ADAMTS-13 deficiency, which has been previously demonstrated its efficacy for TTP. While both caplacizumab and RNA aptamer are approved by the U.S. Food and Drug Administration for the treatment of acquired iTTP, only caplacizumab is currently used clinically [13,14]. Anfibatide was only tested in the preclinical murine model of TTP, which demonstrated its therapeutic efficacy in a Shiga toxin-induced model of TTP [24,26]. Because anfibatide is purified from snake venom [45,46], it may have a limited source of supply and a small scale of production for therapeutic purposes.

Here, we have developed a novel platelet GPIbα antagonist, the first in class humanized monoclonal antibody Fab fragment (ie, CA1001), which has been produced at the industrial scale in the GMP facility. This facilitates the implementation of clinical trials for iTTP and hereditary TTP, as well as other thrombotic disorders such as ischemic stroke. We demonstrate that CA1001 is a potent inhibitor of platelet-VWF interaction under various pathophysiological conditions. Most importantly, CA1001 exhibits preventive and potentially therapeutic effects in a murine model of “TTP-like” syndrome, which is triggered by a pathophysiological stimulus such as H5505, which is released from neutrophils that undergo NETosis during acute and chronic inflammation [26,47–51]. Because of a relatively short half-life of CA1001 (~4 hours), we administered CA1001 with an initial loading dose of 8.0 mg/kg body weight i.v., followed by a s.c. dose of 4.0 mg/kg body weight, similar to the dosing schedule of caplacizumab for therapy of acquired iTTP in humans [13,14]. In our pilot experiment, a single dose of CA1001 (8 mg/kg body weight) did not show a significant protective effect against histone-induced thrombocytopenia (data not shown). Therefore, we changed the dosing schedule to an initial i.v. infusion and s.c. administrations of CA1001, which provided more sustained levels of CA1001 and resulted in significant protection against histone-induced thrombocytopenia and prerenal injury, likely resulting from the rare formation of microvascular thrombosis.

*Adamts-13*^*−/−*^ mice with the C57BL/6J background do not develop spontaneous TTP due to lower levels of plasma VWF compared with other strains of mice, such as CAST/Rk [33]. However, i.v. administration of Shiga toxin 2 [33,52], recombinant VWF [34], or H5505 [30] may trigger the acute onset of “TTP-like” syndrome, primarily manifested by moderate to severe thrombocytopenia and increased levels of LDH and creatinine. Hemolysis, resulting in a reduction of hematocrit and fragmentation of red blood cells, is sometimes observed in murine models [29]. Studies have shown that both Shiga toxin 2 and lysine-histone are able to activate endothelium that releases ultralarge VWF [30,53,54]. These agents may also activate complements, resulting in the formation of membrane attack complexes that damage endothelial surfaces [55,56]. When plasma ADAMTS-13 is severely deficient, Shiga toxin 2 or lysine-histone can trigger the “TTP-like” syndrome in mice. However, the VWF release following the histone challenge is relatively transient due to the short half-life of histone. This explains why platelet counts recover within 48 hours following a single dose of lysine-histone challenge. Additionally, excessive VWF multimers are rapidly cleared from circulation [57]. Nevertheless, CA1001 appeared to show a moderate protective role in *Adamts-13*^*−/−*^ mice against the development of “TTP-like” syndrome (eg, thrombocytopenia and prerenal injury). There was no increase in the BUN/creatinine ratio in mice that received CA1001, suggesting the protective role in renal function. Histologic and immunohistochemical analysis confirmed the presence of rare microthrombi in the liver and kidneys in mice following challenge with lysine-histone. This may reflect the difference in organ sensitivity to an i.v. lysine-histone challenge in mice.

There are some limitations to this study. The TTP phenotype seems to be mild to modest following the histone challenge. The specific binding site of CA1001 on GP1b and the *in vivo* clearance mechanism is not fully characterized.

Nevertheless, this is the first time we have demonstrated that a humanized antibody Fab fragment against platelet GPIbα (ie, CA1001) is a potent inhibitor of platelet-VWF interaction, which inhibits thrombus formation under various conditions. CA1001 exhibits preventive and therapeutic efficacy for a “TTP-like” syndrome in a murine model. Thus, CA1001 may be a valuable candidate considered for the development of a novel therapy for TTP in the future.

## Supplementary Material

1

2

The online version contains supplementary material available at https://doi.org/10.1016/j.jtha.2025.02.009

## Data Availability

The data and key reagents are available for sharing by email request to the corresponding authors.
